# Synergistic Up-Regulation of CXCL10 by Virus and IFN γ in Human Airway Epithelial Cells

**DOI:** 10.1371/journal.pone.0100978

**Published:** 2014-07-17

**Authors:** Karen L. Oslund, Xu Zhou, Boram Lee, Lingxiang Zhu, Trang Duong, Robert Shih, Nicole Baumgarth, Li-Yin Hung, Reen Wu, Yin Chen

**Affiliations:** 1 Center for Comparative Respiratory Biology and Medicine, Department of Internal Medicine, University of California Davis, Davis, California, United States of America; 2 Department of Pathology, Immunology and Microbiology, School of Veterinary Medicine, University of California, Davis, Davis, California, United States of America; 3 Department of Pharmacology and Toxicology, College of Pharmacy, University of Arizona, Tucson, Arizona, United States of America; University of Pittsburgh, United States of America

## Abstract

Airway epithelial cells are the first line of defense against viral infections and are instrumental in coordinating the inflammatory response. In this study, we demonstrate the synergistic stimulation of CXCL10 mRNA and protein, a key chemokine responsible for the early immune response to viral infection, following treatment of airway epithelial cells with IFN γ and influenza virus. The synergism also occurred when the cells were treated with IFN γ and a viral replication mimicker (dsRNA) both *in vitro* and *in vivo*. Despite the requirement of type I interferon (IFNAR) signaling in dsRNA-induced CXCL10, the synergism was independent of the IFNAR pathway since it wasn’t affected by the addition of a neutralizing IFNAR antibody or the complete lack of IFNAR expression. Furthermore, the same synergistic effect was also observed when a CXCL10 promoter reporter was examined. Although the responsive promoter region contains both ISRE and NFκB sites, western blot analysis indicated that the combined treatment of IFN γ and dsRNA significantly augmented NFκB but not STAT1 activation as compared to the single treatment. Therefore, we conclude that IFN γ and dsRNA act in concert to potentiate CXCL10 expression in airway epithelial cells via an NFκB-dependent but IFNAR-STAT independent pathway and it is at least partly regulated at the transcriptional level.

## Introduction

Influenza pneumonia remains a major cause of morbidity and mortality worldwide. Airway epithelial cells are the first line of defense against viral infections in the lung and are instrumental in coordinating the early inflammation leading to an adaptive immune response. CXCL10 (IFN gamma inducible 10 kDa protein, IP10) is a non-ELR CXC chemokine with potent biological effects including monocyte stimulation, natural killer and activated T cell migration, modulation of adhesion molecule expression, inhibition of angiogenesis [1] as well as antimicrobial effects at high concentrations [2].

The role of CXCL10 in viral pneumonia has not been thoroughly characterized but evidence suggests it is important for the migration of NK cells, macrophages, T cells, neutrophils and plasmacytoid dendritic cells into the lung [3], [4]. In a mouse model of RSV infection, antibody-mediated neutralization of CXCL10 resulted in a significant increase in disease symptoms including impaired viral clearance, reduced pulmonary dendritic cell numbers and maturation and a reduction in viral specific CD8(+) T cells [5].

Synergistic up-regulation of CXCL10 has been described *in vitro* in several cell types and in response to different pro-inflammatory molecules but always in conjunction with IFN γ. TNFα and IFN γ induce synergistic levels of CXCL10 in a human endothelial cell line HMEC-1 via an ERK dependent pathway [6], IL-1β and IFN γ in human intestinal epithelial cell lines [7], PDGF and IFN γ in blood derived macrophages [8], TNFα and IFN γ in primary human airway smooth muscle cells and gastric epithelial cells [9], [10], prolactin and IFN γ [11] as well as substance P and IFN γ in human keratinocytes [12] and hyaluronan fragments with IFN γ in mouse macrophages [13]. Interestingly, synergistic induction of CXCL10 has been described with IFN γ in conjunction with HIV-1 in human astrocytes and macrophages and thought to play a role in HIV induced encephalopathy [14], [15]. This marked variety of pro-inflammatory molecules that elicit a synergistic response of CXCL10 in a wide variety of cell types is indicative of a highly conserved and likely biologically important cellular response. However, synergistic induction of CXCL10 in response to influenza and IFN γ in airway epithelial cells has not been previously reported.

In this study, we demonstrate synergistic induction of CXCL10 in well differentiated primary human bronchial epithelial (HBE) cells following influenza virus infection and the treatment with IFN γ. We further demonstrate that this synergy was mediated by the interaction between dsRNA (an intermediate of viral replication) and IFN γ *in vitro* and *in vivo*. In the follow-up mechanistic study, this synergism was found to be transcriptionally regulated as demonstrated by a chimeric promoter reporter gene assay. In addition, it was not dependent of the IFNAR pathway as neither the neutralizing antibody to IFNAR nor the IFNAR deficiency affected the synergism. Finally, NFκB, but not STAT1, appeared to mediate this synergism.

## Materials and Methods

### Culture and treatments of primary human bronchial epithelial (HBE) cells

Human bronchial tissues were purchased from the commercial source (National Disease Research Interchange, Philadelphia, PA) as described before [16], [17]. No tissues from patients diagnosed with lung-related diseases were used. All those lungs were either autopsy leftovers or were rejected for transplant. They were sent to us with arbitrary numerical code. No identity link to the actual patient can be identified. Protease-dissociated HBE cells were plated on Transwell® chambers (Corning) and were maintained in immersed culture conditions until they reached confluence when they were transferred to an air-liquid interface (ALI) culture condition [16], [17]. At air-liquid interface, the cells were maintained in a Ham’s F12/DMEM (1∶1) with the addition of transferrin (5 µg/ml), insulin (5 µg/ml), cholera toxin (10 ng/ml), epidermal growth factor (10 ng/ml), dexamethasone (0.1 µM), bovine hypothalamus extract (15 µg/ml), BSA (0.5 mg/ml) and all-*trans*-retinoic acid (30 nM). Cells were maintained at ALI for 7 days and were placed in basal media devoid of the additives, with the exception of retinoic acid, overnight prior to the experiments. Recombinant IFN γ was purchased from R&D Systems and synthetic double stranded (ds) RNA (i.e. poly I:C) from EMD Biosciences. IFN γ was used at 50 ng/ml and poly I:C at 25 µg/ml. A neutralizing antibody to IFNAR was obtained from US Biologics and used at 2.5 µg/ml [18]. Both IκB kinase and JAK inhibitors were purchased from EMD Biosciences and used at 5 µM. For influenza infection, cells were infected with the influenza virus strain MEM at 1200 HAU.

### Mice

For *in vivo* study, BALB/c mice were purchased from Charles Rivers Laboratories and used at 6–8 weeks of age for intra-tracheal instillations. For primary airway epithelial cell cultures, IFNAR null mice were a kind gift from Dr. Nicole Baumgarth (UCD) and the wild-type controls mice were C57BL/6J. Mice were used at 8–10 weeks of age. All protocols described were approved by the University of California, Davis and University of Arizona, which were responsible for the proper care and use of experimental animals.

### Culture and treatments of primary mouse tracheal epithelial (MTE) cells

The protocol was performed as described previously [19]. Briefly, at the time of necropsy, the chest and cervical region were exposed. A small puncture was placed in the proximal trachea to allow cannulation with sterile 0.86 mm polyethylene tubing (Intramedic Clay Adams) which was secured in place with a 3.0 silk suture. A loose suture was placed at the distal end of the trachea just proximal to the carina. The trachea was dissected free and immediately placed in DMEM at 4°C. And each trachea gently inflated with 0.2% protease through the tracheal cannula after tightening the distal suture. Dissociated epithelial cells were gently harvested by injecting 5 milliliters of cell culture media through the trachea. MTE cells from all tracheas were pooled and re-suspended in cell culture media prior to plating on Transwell© chambers (Corning) coated with Purcol© (Advanced Biomatrix). MTE cells were maintained in submerged conditions until confluent at which time they were kept in air-liquid interface conditions for one week in the presence of 100 nM retinoic acid. For the experiments, MTE cells were treated with 50 ng recombinant murine IFN γ (R and D systems) and/or 25 µg dsRNA for 24 hours.

### Mouse model of synergism

The protocol was performed as described previously [20], [21]. Briefly, BALB/c mice were anesthetized with isoflorane. In dorsal recumbancy, the tongue was gently pulled out, and a pipette tip was gently inserted into their laryngeal region through the oral cavity. 2 mg of dsRNA and/or 5 µg of recombinant murine IFN γ in 50 µl of LPS free PBS was deposited and breathed in. LPS free PBS was used as the control. Mice were kept in an upright position until recovery from anesthesia. Twenty four hours later, mice were euthanized, and lungs were lavaged with 1 ml PBS. All lungs were flash frozen in liquid nitrogen for subsequent RNA isolation.

### RNA isolation and real-time RT-PCR

Total RNA was extracted with TRIzol© reagent (Invitrogen) and cDNA was generated from an equal amount of RNA (1 µg per reaction) by Moloney’s murine leukemia virus-reverse transcriptase (Applied Biosystems) using random hexamers (Invitrogen). SYBR Green Master Mix (Roche Applied Science) and the ABI7900HT Detection System (Applied Biosystems) were used following the manufacturer’s protocol for real-time PCR analysis. The relative mRNA amount of each sample was calculated based on its threshold cycle, Ct, in comparison to the Ct of the housekeeping gene, beta actin or glyceraldehyde 3-phosphate dehydrogenase (GAPDH). The results are presented as 2^−(Ct CXCL10– Ct of housekeeping gene)^ as fold induction over the control condition. The purity of the amplified product was determined as a single peak of the dissociation curve. Throughout the study, there was no observable fluctuation in the Ct values of the housekeeping genes from different treatment conditions. Primers to human and murine CXCL10 were purchased from SA Biosciences. The primer sequences for human beta actin are as follows: forward: TGTGTCCGTCGTGGATCTGA and reverse: CCTGCTTCACCACCTTCTTGAT. The primer sequences for murine GAPDH are as follows: forward: TCCTCCACCTTTGACGCTG and reverse: ACCACCCTGTTGCTGTAGCC.

### CXCL10 ELISA

In preparation for collecting conditioned media from primary human airway epithelial cells, the cells were washed twice with sterile PBS. Fresh media containing IFN γ and/or poly I:C was placed basally with 100 µl of media placed apically. Following a 24 hour incubation, the media was harvested, centrifuged to remove any cellular debris and stored at −80°C. Bronchoalveolar lavage fluid was used for the murine CXCL10 ELISA. Commercially available ELISA kits were used according to the manufacturer’s directions (R&D Systems). Results were normalized to the amount of conditioned media or lavage fluid and expressed as ng/ml or pg/ml.

### Luciferase assay

The construct contains 735 bp upstream of the transcription start site of CXCL10 was cloned into PGL3 luciferase reporter vector (promega). The construct was confirmed by DNA sequencing. Early differentiated HBE cells (7 days) were transfected with CXCL10 promoter-luciferase construct and pRL-TK using Lipofectamine 2000 (Invitrogen) according to the manufacturer’s specifications. pRL-TK was used as the internal control for normalizing transfection efficiency. The empty vector, pGL3 basic was used as a negative control. In brief, cells were plated onto 12 well plates at 80–90% confluency. The next day, cells were washed twice with Opti-MEM (Invitrogen) before transfection. The cells were incubated at 37°C with the mixture of DNA constructs and Lipofectamine 2000 in Opti-MEM for 5–6 hours at which time cell culture media was also added to the cells. Transfected cultures were treated with 50 ng/ml IFN γ and/or 25 µg poly I:C for 24 hours. A dual luciferase reporter assay kit (Promega) was used following the manufacturer’s protocol. For each transfection, the relative firefly luciferase activity was normalized to the renilla luciferase activity.

### Neutralization of IFNAR

2.5 µg/ml of a mouse monoclonal antibody to chain 2 of the human alpha, beta, omega interferon receptor (IFNAR) was added to culture media of cells for 1 hour prior to the start of the experiment. This antibody has previously been documented to neutralize the extracellular domain of the human type I interferon receptor with high affinity at the dose used in this study [18]. IFN γ and/or poly I:C were added to the culture media following the pre-incubation period and with the IFNRA antibody still present. Mouse IgG was used as the control. Following a 24 hour incubation, RNA isolation and qPCR were performed as previously described.

### Statistical analysis

Data are expressed as mean ± SE. Experiments were conducted in triplicate with at least three independent cultures. Group differences were calculated using an Analysis of Variance (ANOVA) followed by a Bonferroni multiple comparison test. Differences were considered significant for p values less than 0.05.

## Results

### Synergistic stimulation of CXCL10 mRNA following influenza virus infection and IFN γ treatment in HBE cells

**Figure 1:**
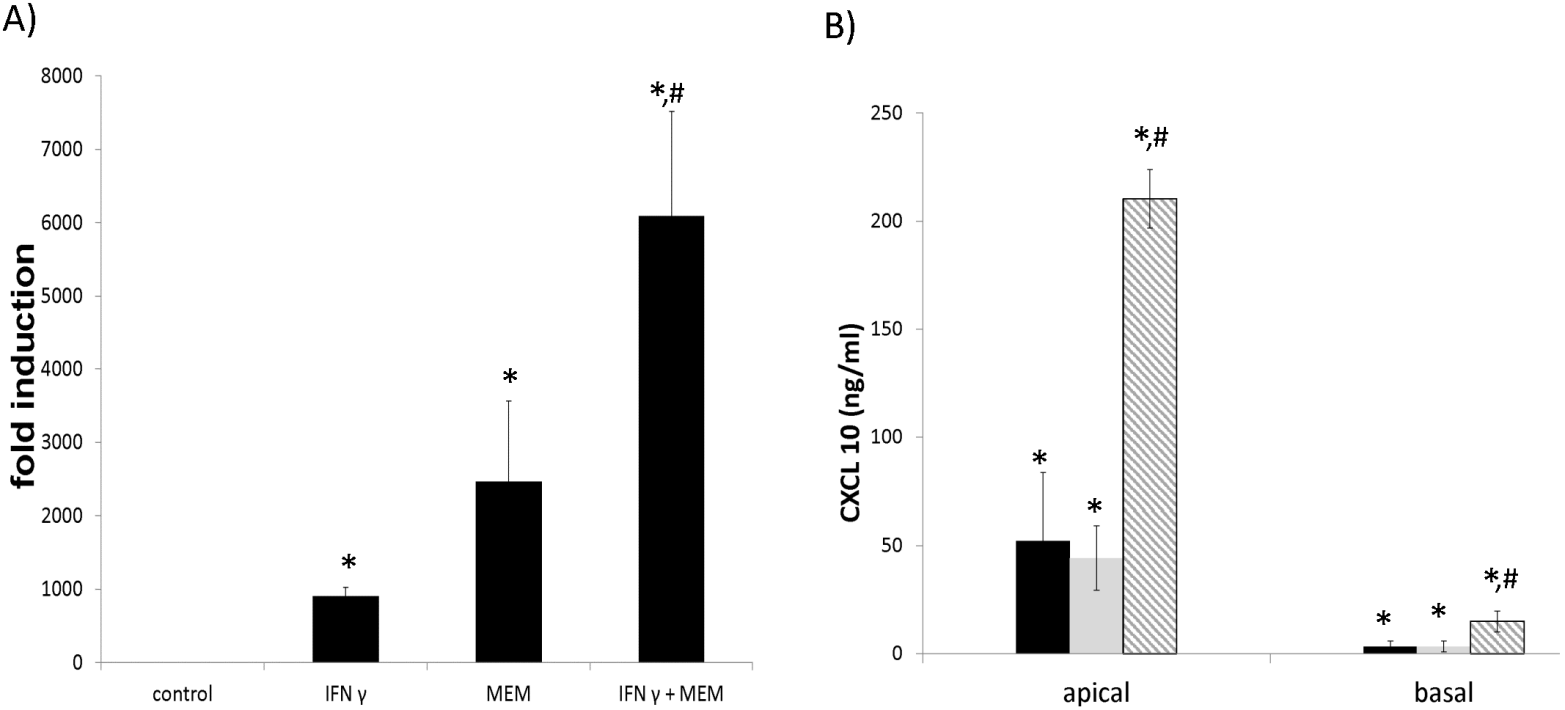
CXCL10 mRNA and protein induction in HBE cells with influenza virus infection. Well differentiated primary HBE cells were treated with 50/ml IFN γ and/or 1200 HAU MEM influenza virus for 24 hours. A) RNA was isolated followed by qPCR analysis. B) Culture media from either apical or basal compartment were collected and analyzed by ELISA assay. Black bars: IFN γ only, grey bars: MEM only, hatched bars: IFN γ+MEM. Triplicate wells were used for each experiment and experiments were repeated at least three times using cultures derived from different donors. *: p<0.05 compared to the control. #: p<0.05 compared to either IFN γ treatment or MEM infection alone.

As shown in Fig. 1A, well differentiated HBE cells demonstrated significant synergistic induction of CXCL10 mRNA following infection with the MEM influenza virus and treatment with IFN γ. CXCL10 induction was also significantly elevated following the combined treatment of MEM and IFN γ treatment as compared to the treatment with IFN γ alone. Consistently, potentiation of CXCL10 protein production by the combined treatments was detected in the cell culture supernatant from HBE cells (Fig. 1B). Apical release of CXCL10 was enhanced in all treatment conditions with more pronounced levels detected in the combined MEM infection and IFN γ treatment (Fig. 1B). Interestingly, significant levels of CXCL10 were also released basally except that they were much lower than the apical secretion. These results demonstrate that influenza virus in combination with IFN γ synergistically induce CXCL10 mRNA and protein production from primary human airway epithelial cells.

### Synergistic stimulation of CXCL10 mRNA and protein by IFN γ and dsRNA in HBE cells

**Figure 2:**
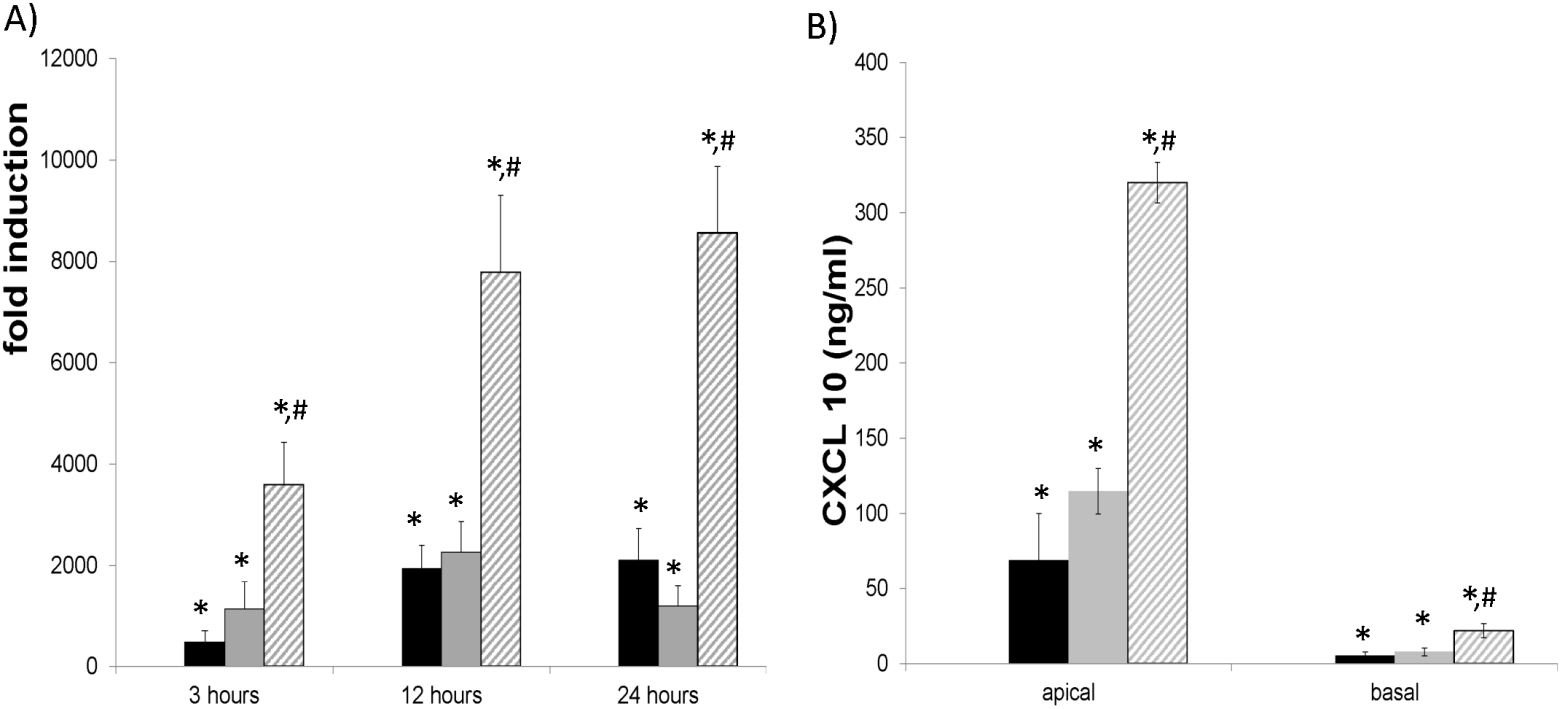
Time-dependent CXCL10 mRNA and protein induction in HBE cells with dsRNA treatment. Well differentiated primary HBE cells were treated with 50/ml IFN γ and/or 25 µg/ml dsRNA. RNA was collected at 3, 12 or 24 hours and analyzed by qPCR. Culture media from either apical or basal compartment were collected at 24 hours and analyzed by ELISA assay. Black bars: IFN γ only, grey bars: dsRNA only, hatched bars: IFN γ + dsRNA. Triplicate wells were used for each experiment and experiments were repeated at least three times using cultures derived from different donors. *: p<0.05 compared to the control. #: p<0.05 compared to either IFN γ or dsRNA treatment alone.

Because viral replication and its intermediate-double stranded (ds) RNA have been shown to mediate many influenza induced phenotypes. We then examined the possibility if dsRNA could synergize with IFN γ in the induction of CXCL10. As shown in Fig. 2A, HBE cells demonstrated synergistic induction of CXCL10 mRNA in a time dependent manner starting as early as 3 hours after treatment. Treatment with IFN γ and dsRNA resulted in significant synergistic induction of CXCL10 mRNA levels at all the time points. Combined treatment for 24 hours resulted in an over 8000 fold induction of CXCL10 mRNA. Consistently, Fig. 2B demonstrates potentiation of CXCL10 protein in cell culture supernatant from HBE cells treated for 24 hours. Apical release of CXCL10 was enhanced in all treatment conditions with more pronounced levels detected in the combined IFN γ and dsRNA treatment. Significant levels of CXCL10 were also released basally, although the levels were much lower than the apical secretions. These results suggest that the synergy between influenza infection and IFN γ treatment may be caused by the interaction between dsRNA- and IFN γ-mediated signaling pathways.

### Synergistic stimulation of CXCL10 mRNA and protein in vivo

**Figure 3:**
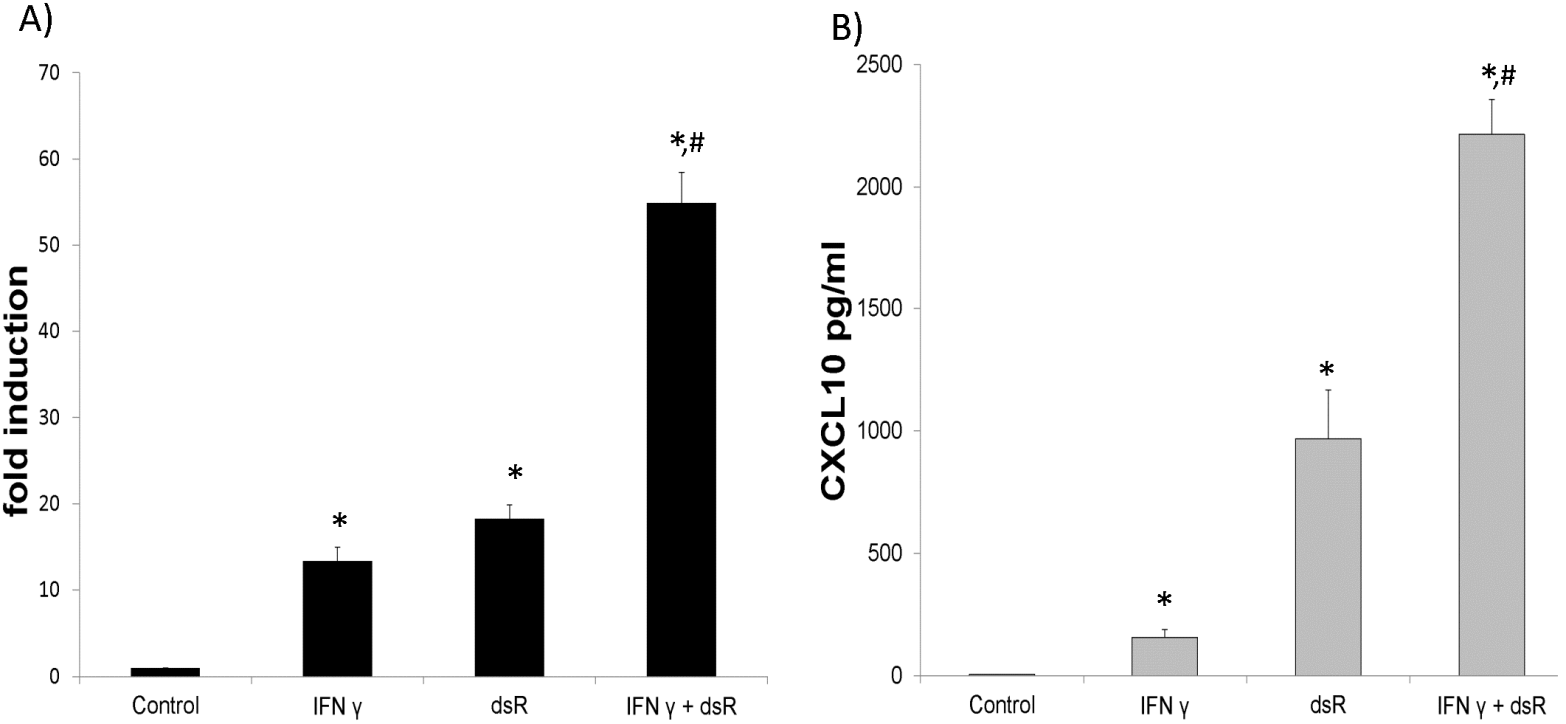
CXCL10 mRNA and protein induction in BALB/c lungs. BALB/c mice were treated with 5 µg/ml IFN γ and/or 2 mg dsRNA (dsR). Twenty four hours later, bronchoalveolar lavage fluid and trachea and lungs were harvested. N = 3 per condition. Values are presented as the mean ± SE. A) Total RNA was isolated from the trachea and lungs followed by qPCR. B) CXCL10 protein was assayed in BAL fluid using an ELISA. Samples were normalized to BAL returned volume. *: p<0.05 compared to the control. #: p<0.05 compared to either IFN γ or dsRNA treatment alone.

To determine whether IFN γ and dsRNA synergistically induce CXCL10 *in vivo*, we performed intra-tracheal delivery of IFN γ and dsRNA in BALB/c mice and examined CXCL10 mRNA and protein levels. As shown in Fig. 3A, dsRNA alone induced a significant increase in CXCL10 mRNA, and IFN γ and dsRNA treatment together synergistically induced CXCL10 mRNA 54 fold in mouse lung. Likewise, Fig. 3B demonstrates a significant induction of CXCL10 protein in bronchoalveolar lavage fluid after treatment with dsRNA and synergistic induction following IFN γ and dsRNA treatment. These results demonstrate synergistic induction of CXCL10 mRNA and protein *in vivo* following IFN γ and dsRNA treatment.

### Synergistic stimulation of CXCL10 gene by IFN γ and dsRNA is independent of the type I interferon pathway

**Figure 4:**
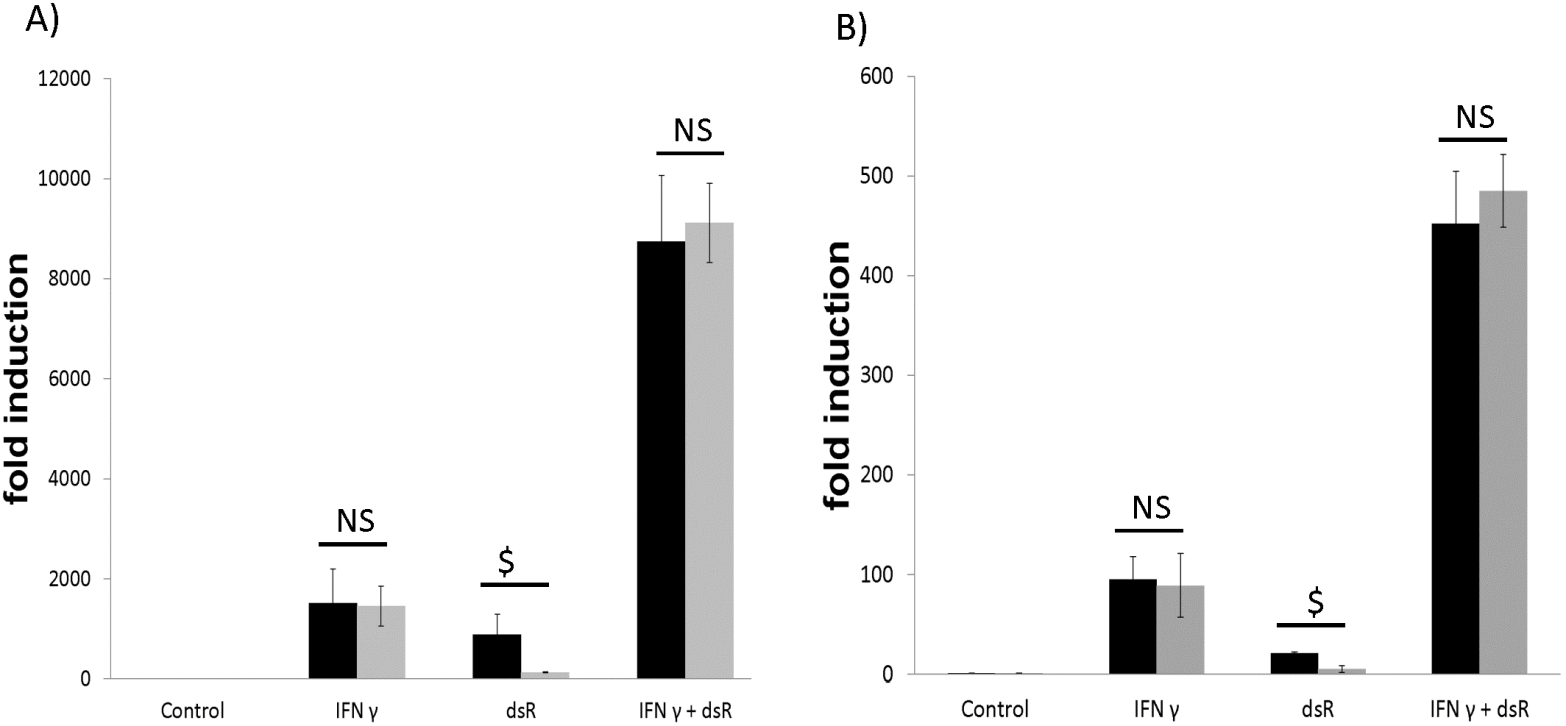
The synergism between IFN γ and dsRNA(dsR) was independent of IFNAR. A) HBE cells were treated with 2.5 µg/ml of either an isotype control antibody (black bar) or a monoclonal neutralizing antibody IFNAR (Grey bar) for one hour prior to the addition of 50 ng/ml IFN γ and/or 25 µg/ml dsRNA for 24 hours. RNA was isolated followed by qPCR. NS: not significant. $: p<0.05. isotype control antibody vs. IFNAR. B) Primary MTE cells isolated from either wild-type (black bar) or IFNAR deficient (grey bar) mice were grown in vitro and treated with 50 ng/ml IFN γ and/or 25 µg/ml dsRNA for 24 hours. RNA was isolated followed by qPCR. NS: not significant. $: p<0.05. wild-type vs. IFNAR deficient. Triplicate wells were used for each experiment and the experiment was repeated at least three times.

Because dsRNA is known to induce the type I interferon pathway, we investigated the involvement of this pathway by using a neutralizing antibody to human IFNAR. Fig. 4A demonstrates that the IFNAR neutralizing antibody did not affect synergistic induction of CXCL10 mRNA in HBE cells, although it did repress dsRNA-induced CXCL10. To confirm this result, we isolated MTE cells from IFNAR null mice, which were completely lack of IFNAR expression. Fig. 4B demonstrates that the lack of IFNAR did not affect the synergistic induction of CXCL10. Interestingly, induction of CXCL10 by dsRNA alone was significantly impaired in the MTE cells from IFNAR null mice as compared to the cells from wild-type mice. Taken together, these results demonstrate that synergistic induction of CXCL10 mRNA was independent of the type I interferon receptor in both human and mouse airway epithelial cells. In contrast, dsRNA-induced CXCL10 appeared to depend on the type I interferon receptor, suggesting that the synergy may be mediated via a different pathway.

### The synergy between IFN γ and dsRNA on CXCL10 promoter

**Figure 5:**
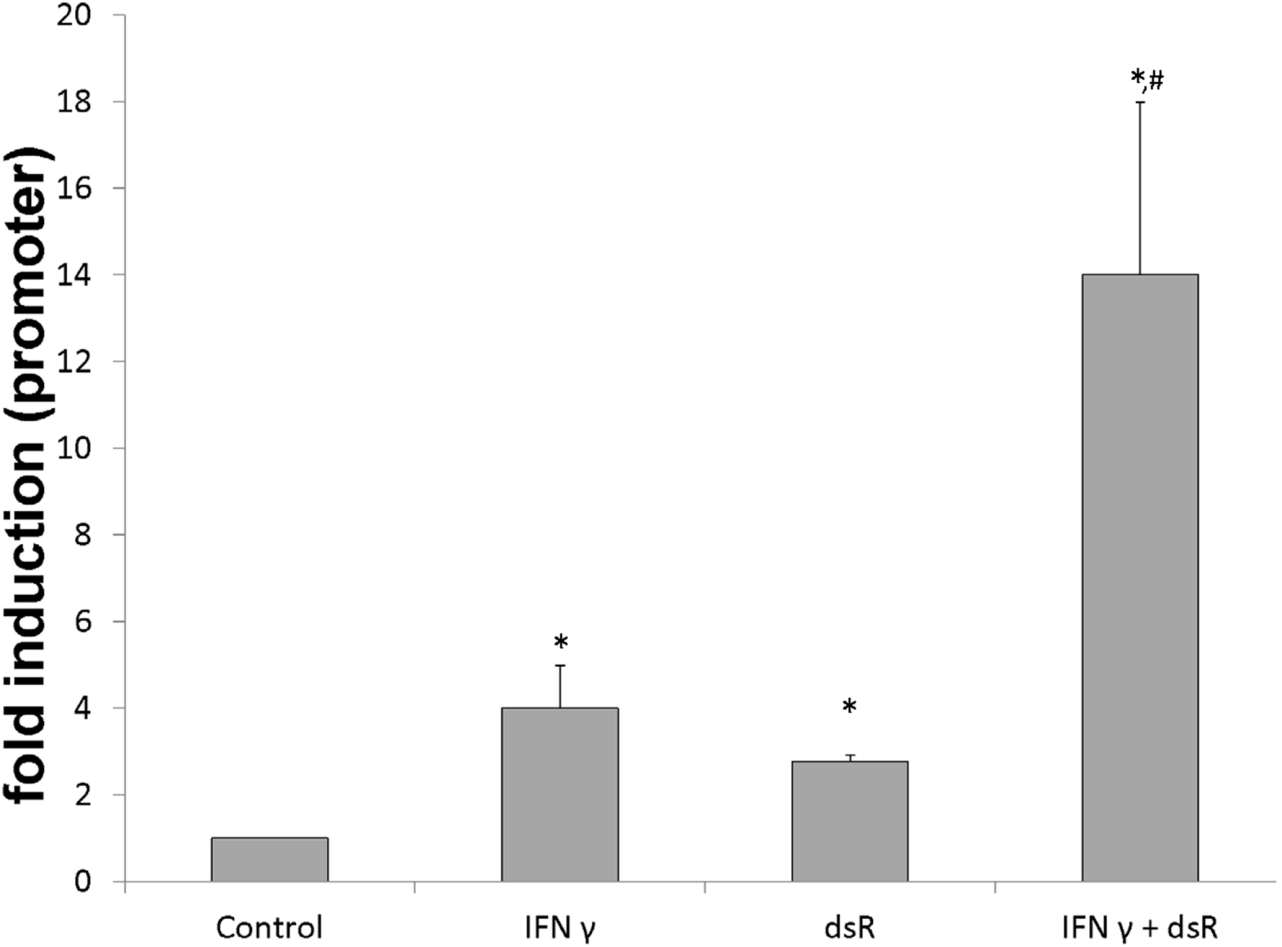
CXCL10 promoter constructs and luciferase assay in HBE cells. CXCL10 promoter-reporter gene activity in response to 50 ng/ml IFN γ and/or 25 µg/ml dsRNA (dsR) for 24 hours. Triplicate wells were used for each experiment and experiments were repeated at least three times. Values are expressed as the mean ± SE. *: p<0.05 compared to the control. #: p<0.05 compared to either IFN γ or dsRNA treatmen alone.

Because of the rapid elevation of CXCL10 by *IFN γ and/or dsRNA* (Fig. 2A), we tested the possibility if this synergy occurred at transcriptional level by transient transfection of the HBE cells with a CXCL10 promoter/luciferase chimeric construct containing 735 bp of the upstream regulatory region of CXCL10. Fig. 5 demonstrates the combined treatment of IFN γ and dsRNA significantly increased the luciferase reporter gene activity as compared with the treatment of IFN *γ* or dsRNA alone. These results support that the synergism occurred at least partially through a direct transcriptional mechanism and the proximal 735 bp region of the CXCL10 promoter was critical for the induction.

### Synergistic stimulation of CXCL10 was mediated by NFκB pathway

**Figure 6:**
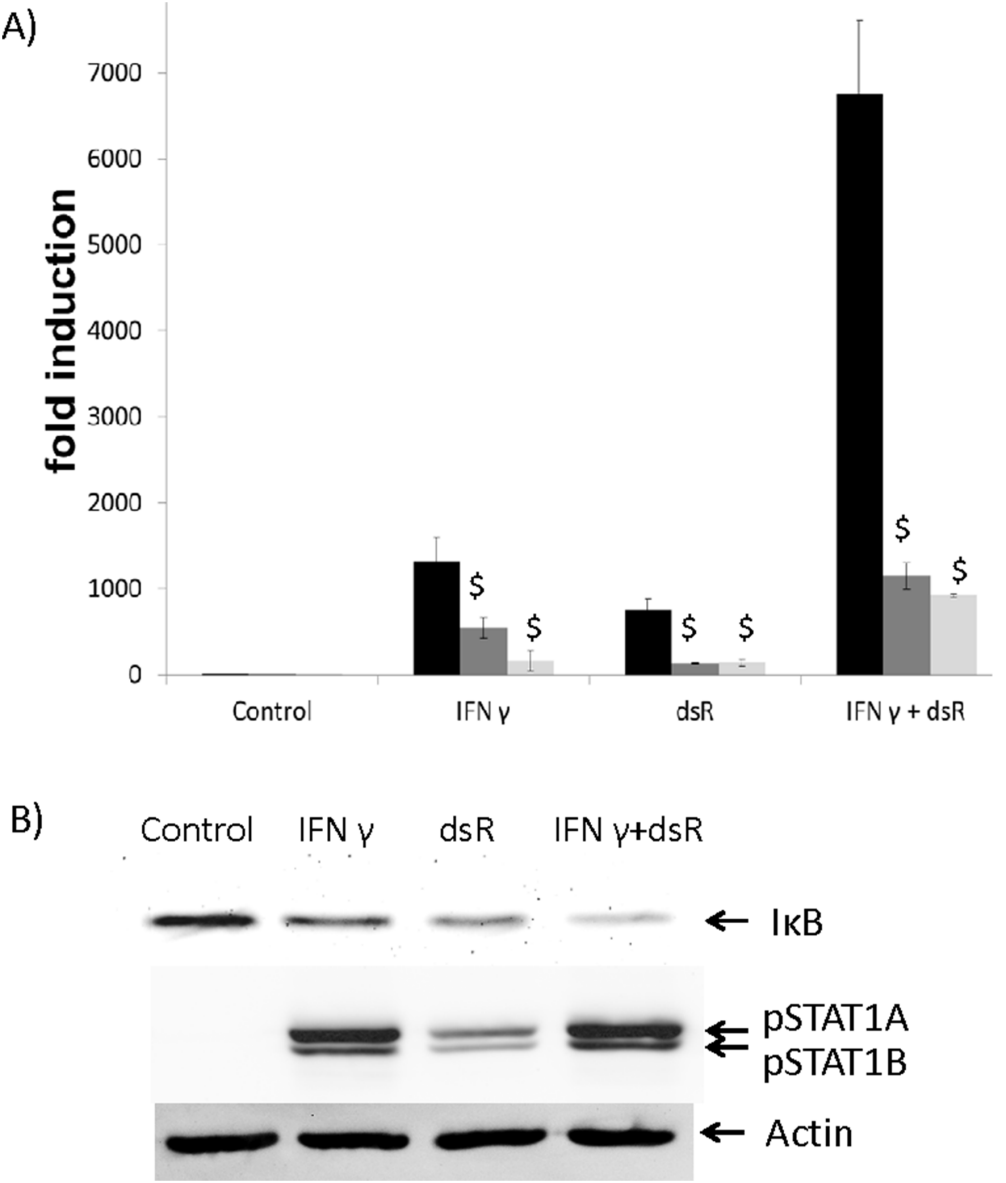
NFκB-dependent signaling is responsible for the synergism between IFN γ and dsRNA (dsR). A) Well differentiated primary HBE cells were treated with 50 ng/ml IFN γ and/or 25 µg/ml dsRNA for 24 hours in the presence or absence of IκB kinase or JAK inhibitor. RNA was isolated followed by qPCR. Solvent Control: black bar; IκB kinase inhibitor: grey bar; JAK inhibitor: light grey bar. $: p<0.05. Inhibitor treatment vs. solvent control. B) Cells were treated with 50 ng/ml IFN γ and/or 25 µg/ml dsRNA for 3 hours. Cellular protein was collected and analyzed by western blot analysis. Actin was used as a loading control. pSTAT1: phosphorylated STAT1. This is the representative image from three replicates.

Because both NFκB and ISRE sites were present in this cloned CXCL10 promoter region, we further tested *NFκB* and STAT pathways in this synergism as both pathways have been shown to be responsible for CXCL10 gene expression. Indeed, the specific inhibitor targeting the kinase either upstream of NFκB (IκB kinase) or STAT1 (JAK) significantly blocked CXCL10 induction by IFN γ, by dsRNA, or by the combined treatment (Fig. 6A), which confirms the indispensable role of both pathways in regulating CXCL1 expression. When the signaling pathway was further examined, the treatment of IFN γ or dsRNA alone was found to induce robust degradation of IκB, a surrogate of the activation of *NFκB* pathway which is initiated by the reaction catalyzed by IκB kinase. And the combined treatment resulted in much greater IκB degradation as compared to the single treatment (Fig. 6B). In contrast, despite the activation of STAT1A and STAT1B by these treatments, no synergism was observed (Fig. 6B). Therefore, the synergism of IFN γ and dsRNA on CXCL10 expression was mediated by NFκB pathway.

## Discussion

Airway epithelial lining is the first defense against respiratory viral infection. In this study, we seek to understand the modulation of an important chemokine-CXCL10 by the combined effects of IFN γ and viral infection. This is the first report documenting the observation and mechanism underlying the synergism between IFN γ and viral infection (or dsRNA treatment) in airway epithelial cells. IFN γ is a type II interferon and oftentimes elevated in the context of viral infection. It is generally accepted that direct innate antiviral defense is mediated by type I IFN. Indeed, we and others have shown that dsRNA (or virus)-induced epithelial derived type I IFN plays critical role in the airway antiviral defense [16], [21], [22]. In the present study, we demonstrate the synergy between dsRNA- and IFN γ-induced signaling in the regulation of CXCL10. Interestingly, although dsRNA-induced CXCL10 depended on type I IFN pathway, the synergy between dsRNA and IFN γ was completely independent of it in both human and mouse epithelial cells. This lack of dependence was not due to the remaining residue of the IFNAR functionality in the antibody neutralization assay, because the epithelial cells from IFNAR deficient mice used in the Fig. 4A still preserved the synergistic response despite the loss of entire IFNAR expression. Thus, in the presence of IFN γ, dsRNA (or virus)-induced signaling is very likely to be altered, which emphasizes the importance of studying the pathway crosstalk as demonstrated in the present study.

Previous studies have demonstrated that IFN γ in conjunction with different pro-inflammatory molecules leads to a synergistic and dramatic induction of CXCL10 in a variety of cell types including keratinocytes, macrophages, endothelial cells and smooth muscle cells [6], [8], [9], [12]. Several of these studies have demonstrated that the transcription factor, NF-κB, is involved in CXCL10 induction in a variety of systems [7]–[9]. In contrast, very few studies have demonstrated that CXCL10 induction is dependent on an ISRE site in the promoter region. Kanda and co-workers found substance P and IFN γ induced synergism of CXCL10 in human keratinocytes was dependent on an ISRE site and two NF-κB sites in the CXCL10 promoter located −210 to −221 of the transcription start site [12]. In addition, Majumder and co-workers found evidence that IFN γ and TNF α act in synergy via p48 complexes with STAT-1α binding to this same ISRE site in the CXCL10 promoter of human fibrosarcoma lines [23]. Evidence that similar synergism occurs at the transcriptional level in mouse cells has been demonstrated in a small number of *in vitro* studies [13], [24]. Using the murine fibroblast NIH 3T3 cell line, Ohmoir and co-workers established that an ISRE site and one of two NF-κB sites in a 243 bp fragment flanking the transcription start site of the murine CXCL10 gene were critical for IFN γ and TNF α induced synergy [24]. Our study has further extended these studies to the airway epithelial system in the context of viral infection, and the results apparently support the importance of NFκB.

The present study demonstrates the significant contribution of CXCL10 from epithelial cells, which is consistent with the emerging role of active defense by these cells in the respiratory viral infection. CXCL10 secretion by HBE cells appeared to be polarized. The treatment with IFN γ, dsRNA and combined treatments resulted in a progressively enhanced secretion of CXCL10 mainly from apical surface. In contrast, the basal secretions of CXCL10 protein in these cultures were much less. The gradient between the apical and basal compartments may have important implications *in vivo* leading to enhanced emigration of CXCR3 positive effector cells (primarily CD8+ T cells) to the airway epithelial microenvironment.

Although dsRNA has been used extensively as a synthetic dsRNA to model viral infections, its stabilized derivatives are also being investigated for clinical use as an immunomodulatory agent for use as vaccine adjuvants in anti-viral treatment and cancer therapies [25]–[28]. While dsRNA was used in this study to simulate a viral infection, it is important to note that it, in and of itself, can also be involved in synergistic induction of CXCL10 in airway epithelial cells. This fact could be an important consideration in the treatment of patients with pre-existing diseases in which IFN γ is part of the pathogenesis such as viral infections.

In summary, we demonstrate IFN γ and the influenza virus synergistically induce CXCL10 in human airway epithelial cells. This synergism is likely to be mediated by dsRNA-induced signaling both *in vitro* and *in vivo*, which is independent of the type I interferon receptor pathway. Furthermore, we demonstrate the 735 bp proximal region of the CXCL10 promoter plays a critical role in regulating this synergistic induction. This region of the CXCL10 promoter contains punitive ISRE and NFkB transcription factor binding sites. Therefore, further study has been carried out to demonstrate the involvement of NFkB, but not STAT1, in this synergism. This capacity for airway epithelial cells to markedly up-regulate CXCL10 likely has important consequences for CD8+ T cell and NK cell migration to the airway epithelial microenvironment following influenza virus infection.
